# My Diabetes My Way: supporting online diabetes self-management: progress and analysis from 2016

**DOI:** 10.1186/s12938-019-0635-4

**Published:** 2019-02-08

**Authors:** Scott Gordon Cunningham, Massimo Brillante, Brian Allardice, Nicholas Conway, Ritchie Robert McAlpine, Deborah Jane Wake

**Affiliations:** 1 School of Medicine, University of Dundee, MacKenzie Building, Kirsty Semple Way, Dundee, Scotland DD2 4BF UK; 2 Usher Institute of Population Health Sciences and Informatics, University of Edinburgh, Edinburgh, Scotland UK

**Keywords:** Diabetes, Self-management, Self-care, Personal health record, Electronic record, Long-term condition, Online, eHealth

## Abstract

**Background:**

My Diabetes My Way (MDMW) is the National Health Service (NHS) Scotland website for people with diabetes and their carers. It consists of an interactive information website and an electronic personal health record (ePHR) available to the 291,981 people with diabetes in Scotland. We aimed to analyse the demographic characteristics of current registrants and system usage and activity during 2016.

**Methods:**

We analysed system audit trails to monitor user activity and page accesses on the information website, and logins and activity within the ePHR. The ePHR contains data from SCI-Diabetes, NHS Scotland’s flagship diabetes record, sourcing data from primary and secondary care, specialist screening services and laboratory systems. We reviewed patient registration characteristics to collate demographic data for the MWDH cohort, then compared this to aggregate data published in the 2016 Scottish Diabetes Survey. The Scottish Diabetes Survey is an annual population-based report detailing diabetes statistics for the whole diabetes population in NHS Scotland.

**Results:**

The MDMW information website received an average of 101,382 page accesses per month during 2016 (56.9% increase from 2015; n = 64,607). ePHR registrants were more likely to be younger (p < 0.001) and have an ethnicity of “white” (p < 0.001) than the background diabetes population. At the end of 2016, 11,840 people with diabetes had accessed their personal clinical information (58.6% increase since end 2015; n = 7464). During 2016, an average of 1907 people accessed their records each month (48.3% increase from 2015; n = 1286).

**Conclusion:**

My Diabetes My Way is a useful tool aid to diabetes self-management. The service is unique in offering records access to a national population, providing information from all relevant diabetes-related sources, rather than a single silo. MDMW supports the diabetes improvement, self-management, healthcare quality and eHealth strategies of the Scottish Government. The service also has potential to be adapted to work with other clinical systems and conditions.

## Background

In 2008, a report by Diabetes UK estimated that diabetes accounted for around 10% of National Health Service (NHS) expenditure [1], equating to £9 billion per year, or £1 million every hour. This was double a 2001 estimate [2], showing the impact of a rising prevalence across the UK. A subsequent report in Diabetic Medicine [3] predicts NHS annual spending on diabetes will increase from £9.8 to £16.9 billion by 2035, reaching 17% of the entire NHS budget. The challenge of supporting self-management in the expanding population of people with diabetes can be assisted through the use of technology, for example, by improving learning and education, much of which can be facilitated electronically [4]. The Diabetes Improvement Plan [5], eHealth Strategy [6] and the Healthcare Quality Strategy for NHS Scotland [7] emphasise the importance of “putting people at the heart of the NHS”, with high quality, evidence-based and patient-focused care. In reality, healthcare provision is organised around healthcare staff availability with little support between infrequent clinic appointments.

Internet-based self-management support and electronic personal health records (ePHRs) have the potential to move the balance of power from healthcare providers to healthcare users and reduce the burden of care by engaging patients in managing their own health and illness. Most existing ePHR systems focus on, and present data residing on single silos (e.g. primary care systems, hospital clinic records). Until the development of My Diabetes My Way (MDMW), there have been no systems worldwide offering a fully population-based, focused “shared electronic record” for diabetes.

**Fig. 1:**
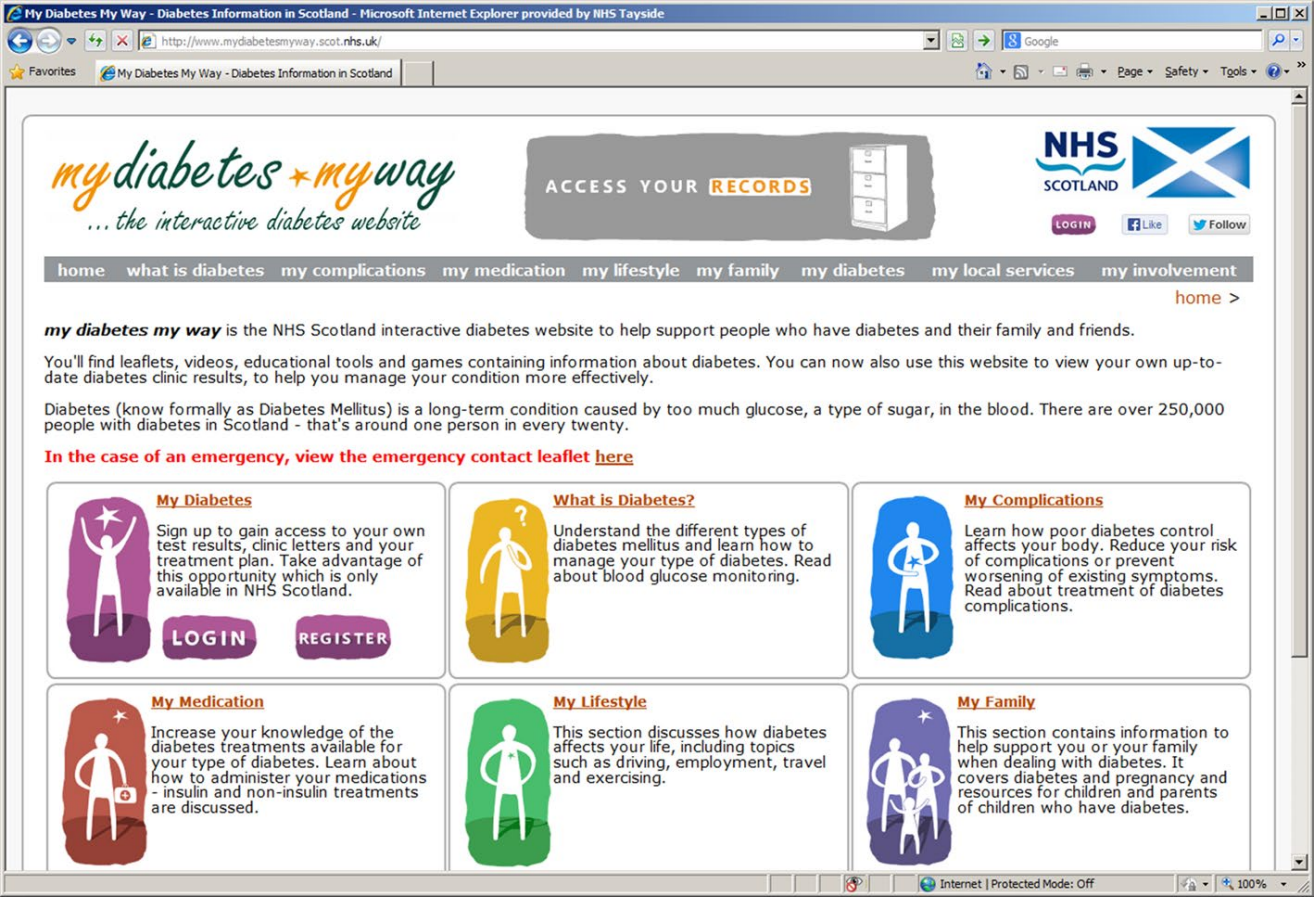
MDMW home page

My Diabetes My Way (MDMW) [8] is the NHS Scotland interactive website for people with diabetes, their friends, family and their carers. It contains a variety of validated multimedia resources aimed at improving self-management. These resources include traditional information leaflets, interactive educational tools, videos describing complications and testimonials from people talking about their experiences with health services. Content covers all aspects of diabetes, including descriptions of the various diabetes types, medication management and administration, diet and exercise, complications and many more (see Fig. 1).

In addition to this general information and advice, MDMW offers its users access to their clinical data via its novel ePHR. This service is available to the 291,981 [9] people with diabetes in Scotland. In order to enrol, a patient must have their demographics validated, verify their email address and consent to their data being made available to them online. The ePHR uses data from SCI-Diabetes [10], NHS Scotland’s flagship diabetes electronic medical record. SCI-Diabetes holds data sourced from primary and secondary care, specialist screening systems (retinopathy screening, podiatry, etc.) and laboratories. The dataset includes diagnostic information, demographics, process outcomes, screening results, medication and clinical correspondence. The system provides a more complete overview of diabetes than would be available from any single data source, such as an isolated primary care or hospital clinic database.

**Fig. 2:**
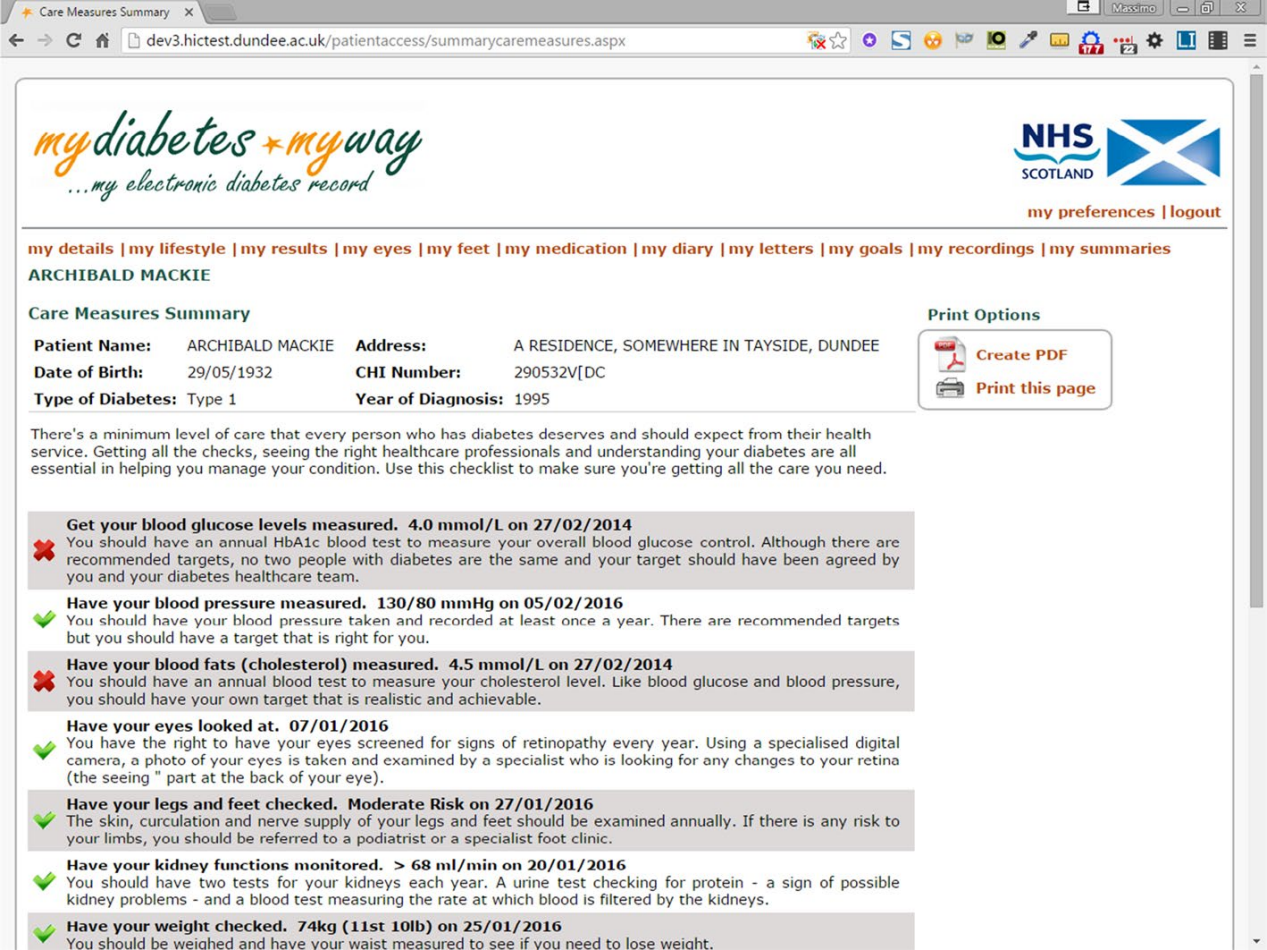
Screenshot: 15 healthcare essentials

The MDMW ePHR takes a subset of data from SCI-diabetes, focusing on key diabetes indicators, such as HbA1c, blood pressure, body mass index, etc. Descriptive text is available alongside these data items, explaining each assessment, detailing why data are recorded and what normal range values are. Further educational materials are presented alongside clinical results and are tailored to those using the service. For example, foot care advice is based on the patient’s recorded foot risk assessment category. History graphs and tables allow individuals to track changes over time for the full duration of their clinical record, from multiple electronic data sources. MDMW aims to provide highly tailored information for users allowing them to make best use of consultation time. For example, Fig. 2 highlights tests that are overdue, linking to the Diabetes UK “15 Healthcare Essentials” campaign [11]. Patients can manually enter home-recorded information (weight, blood pressure, etc.), or automatically upload blood glucose results. These features allow people to take control of their diabetes and become more empowered to enhance their self-management and care.

This study aims to analyse characteristics of service registrants, usage and activity during 2016.

## Methods

My Diabetes My Way system user audit logs were analysed to monitor page accesses and user ‘clicks’ on the general information website. These audit logs capture data regarding pages viewed and the date and time of access, thereby supporting analysis to quantify the generic information and interactive content that was most popular with service users during 2016.

The demographic characteristics of those registering to access their MDMW ePHR were then analysed. This analysis quantified the gender, age, type and duration of diabetes, deprivation status (using the Scottish Index of Multiple Deprivation [12]) and ethnicity of registrants. A comparison was then made with the background diabetes population, held on SCI-diabetes and referenced in the Scottish Diabetes Survey [9]. The Scottish Diabetes Survey is an annual population-based report for NHS Scotland that quantifies incidence, prevalence, demographic and diagnostic characteristics, thereby allowing national reference figures for comparison with the MDMW cohort.

Finally, user activity within the MDMW ePHR by each active user was analysed, using system audit logs. These logs contain data such as user logins, pages viewed and date and time of each event within the system. These logged data made it possible to determine ongoing levels of user engagement following initial system access and highlight the types of clinical data that were most frequently reviewed, the types of summary reports that were most frequently accessed and the most popular user actions (e.g. data entry, etc.).

Data were analysed using IBM SPSS Statistics 22.0, with CHI squared tests used to compare variables from the MDMW population with the background diabetes population for Scotland, references in the Scottish Diabetes Survey.

## Results

The MDMW information website received a total of 1,216,583 page accesses during 2016 (101,382/month), covering educational and learning content. They include general website navigation and the accessing of text, video and interactive resources, user feedback and content searches. The monthly average of 101,382 page accesses during 2016 shows a 56.9% increase from 2015 (n = 64,607). Text- and video-based resources were accessed 233,190 times by users, with content covering ‘HbA1c’, ‘Sick Day Rules’, ‘Healthy Eating and Your Diabetes’, ‘Hypoglycaemia’ and ‘Blood Sugar Testing’ proving some of the most popular (see Table 1).

**Table 1:** Top 10 most popular text- and video-based resources

Page	n	%
HbA1c explained	20,316	8.7
Patient testimonial videos	17,467	7.5
Sick day rules for type 1 diabetes	12,304	5.3
Healthy eating and your diabetes	11,921	5.1
Hypoglycaemia	11,825	5.1
Childhood diabetes video	11,693	5.0
Sick day rules type 2 diabetes	10,028	4.3
Insulin profiles	8483	3.6
Blood sugar testing	8354	3.6
Pregnancy and diabetes video	6008	2.6

**Fig. 3:**
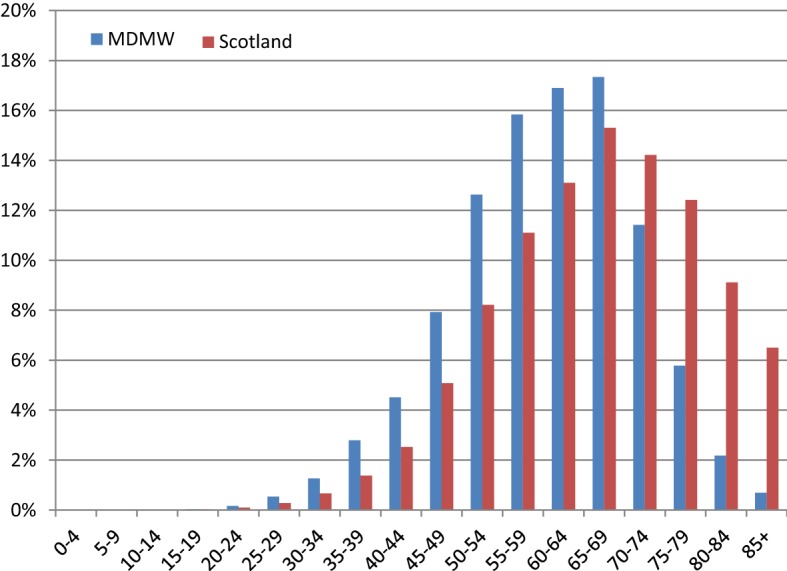
MDMW registrants by age—type 2 diabetes

At the end of 2016, 24,635 people with diabetes in Scotland (8.4% of background population referenced in the Scottish Diabetes Survey) had registered to access their records. 14,325 (58.2%) of these registrants were male and 10,310 (41.9%) were female; 8534 (34.6%) had type 1 diabetes; 15,686 (63.7%) type 2 diabetes; 63 (0.3) pre-diabetes; 303 (1.2%) other types of diabetes and 49 (0.2%) had a diabetes type that could not be determined. System registrants were generally younger than the background population of people with diabetes. This is particularly significant in the type 2 patient group (Fig. 3), where the age distribution between the two populations are significantly different (p < 0.001) when compared with data reported in the 2016 Scottish Diabetes Survey [9]. This shows that in each category of type 2 patients below the age of 70, there are a higher percentage of users in the MDMW cohort than there are in the background population.

Table 2 shows a comparison of the ethnicity of MDMW registrants compared to the background diabetes population. The results shows that the distribution of ethnicity between the two populations are significantly different (p < 0.001). For example, 80.5% of MDMW registrants are recorded as having “White” ethnicity, compared to 75.2% in the general diabetes population. Table 3 details the breakdown of MDMW registrants by deprivation category. The results show that those living in least deprived area (22.5%) are more likely to register to access the service than those in the most deprived area (16.4%).

**Table 2:** Ethnicity of patients: MDMW registrants vs Scotland’s diabetes population

	MDMW	%	Scotland	%
A—white	19,829	80.5	217,019	75.2
B—mixed or multiple ethnic groups	531	2.2	7025	2.4
C—Asian, Asian Scottish or Asian British	442	1.8	9061	3.1
D—African, Caribbean or Black	71	0.3	1117	0.4
E—other ethnic group	216	0.9	1519	0.5
Not recorded/not known	3546	14.4	52,886	18.3
	24,635	100	288,629	100

**Table 3:** Deprivation category of MDMW registrants

SIMD	MDMW	%
Not known	469	1.9
1 (most deprived)	4034	16.4
2	4525	18.4
3	4882	19.8
4	5191	21.1
5 (least deprived)	5534	22.5
	10,577	100

By the end of 2016, 11,840 patients (48.1% of registrants) had completed the enrolment process and logged into access their records (58.6% increase since end 2015; n = 7464). Levels of engagement remain high amongst active users: 4750 (40.1%) logged in at least once during the final 3 months of 2016; 6469 (54.6%) from July to December; 7671 (64.8%) from April to December and 8423 (71.1%) during the full calendar year. There were 74,686 total logins during 2016 (average = 8.8/active user; median = 4). During 2016, an average of 1907 people accessed their records each month (48.3% increase from 2015; n = 1286). Audit trails show 844,729 total page views during 2016 (100.3/patient). The ‘test results’ screen was the most popular summary section of the website (158,874 accesses, 18.9/patient). The most utilised longitudinal history graph was blood pressure, with 13,874 accesses (1.6/patient).

**Fig. 4:**
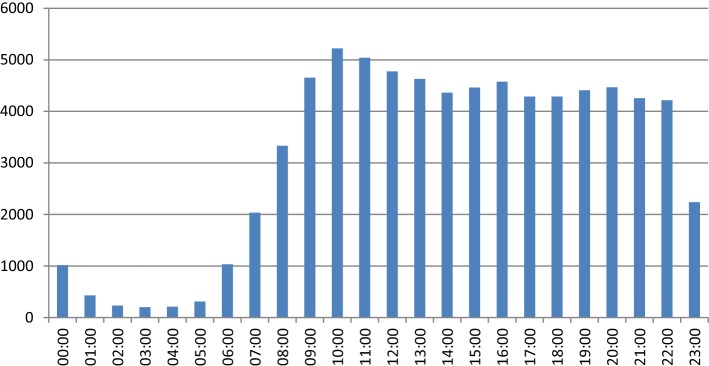
Login by hour of day

**Fig. 5:**
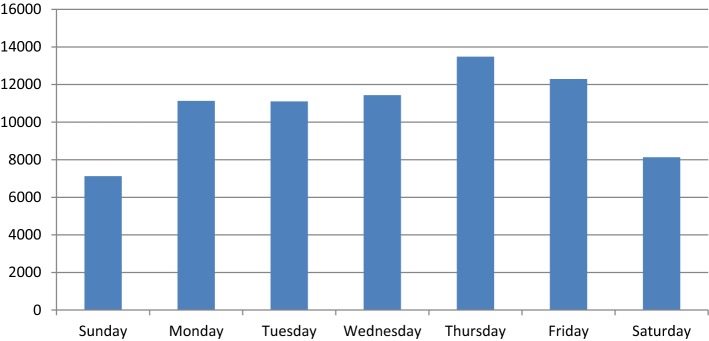
Login by day of week

Login analysis showed that very little activity generally occurs after midnight and before 7am as shown in Fig. 4. Peaks in activity do however occur throughout the day, with increases shown from late morning through midday and again between 4 and 5 pm. Similarly, variation can be observed by day of the week (Fig. 5), with weekends, showing considerably less traffic than weekdays, with a peak of 13,480 during 2016 on Thursday’s.

## Discussion

Prior to the introduction of MDMW, many patients only had access to information and results given to them, often verbally, during clinic consultations. They had no way to look up results themselves or remind themselves of discussions, previous screening, lab or clinic-recorded information that may be useful for self-management and care planning. While some patients may have phoned their doctor for results, or kept paper or electronic notes, these records were likely to remain static, inconsistent and sporadic without significant effort. MDMW provides standardised access to these records through a web-based platform that can be accessed through a variety of devices, from traditional desktop PC, to mobile and tablet devices. This means that any time of the day or night and whenever suitable, the individual can log into access their results or recent assessments.

Despite these benefits, the results highlight some limitations. Firstly, in comparison to the background diabetes population, uptake and usage remains very low, with only 8.4% of patients having registered. Much of this is likely to be due to lack of awareness rather than lack of interest, given that one general practitioner was able to recruit 23.4% of their caseload within 2 weeks of writing to them [13]. It is essential that future activities and front-line services work to advertise the service to patients through mailout campaigns, structured education courses, posters, leaflets and any other available means. Work continues to increase levels of uptake and awareness amongst patients and professionals and we will continue to analyse and assess potential barriers to uptake [14].

The results show that those registering to access MDMW have a consistent gender split when compared with the background population. However, registrants are generally younger, with 34.6% having type 1 diabetes, compared to the NHS Scotland prevalence of 10.9%. This disproportionally high number of type 1′s registering may also be due to younger age, higher levels of diabetes engagement and existing use of technology for diabetes management. Awareness activities in secondary care are more proactive than those in primary care, meaning that people with type 1 diabetes are more likely to see advertising materials at their more frequent appointments, due to their main place of treatment being secondary care. Another possible explanation is that those with perceived poorer outcomes or higher risk of complications are more proactive in finding ways to minimise effects of the condition. There is further scope to extend the existing analyses to include geospatial mapping based on broadband provision and urban/rural divide.

A significant proportion of registrants have a reported ethnicity of ‘White’ and its sub-categories. This indicates that further work is required to engage with minority ethnic groups when compared to the background population. At the present time, while available to all, the MDMW ePHR is only available in English and discussions are underway with NHS24 [15] and the Minority and Ethnic Sub-group of the Scottish Diabetes Group [16] to enhance the content and support for various languages. Issues surrounding literacy factors [17], access to technology, social factors and poor health prioritisation remain barriers requiring pragmatic solutions that extend beyond diabetes to general uptake of digital public services. In the meantime, developments are progressing in the form of patient-configurable preferences that will allow them to select the complexity of the information shown alongside their record.

The analysis shows that those in less deprived areas are more likely to register to use the service than those from more deprived areas. This may be due to availability of access to appropriate technology (computers or mobile devices), or lack of skills to make use of the service, indicating that socio-economic status plays a factor in engagement. We would anticipate that usage amongst disadvantaged groups will improve as the use of apps and digital services to access healthcare continues to become more routine.

It is not surprising to find that blood pressure and test results are the most popular screens viewed by patients, given that these screens contain data presenting current control. It is promising to see that monthly usage increased by nearly 50% compared to data from 2015. User engagement remains high, with 71.1% of those who have ever accessed the service logging in during 2016. The difference between the average and median number of logins shows that some patients access MDMW considerably more than others. Further analysis is required to determine if this is related to diabetes type and frequency of appointments. It will be interesting to monitor these and other trends as usage and the number of patients increases.

When analysing time of login, there are clear and unsurprising trends showing the most popular periods during the course of a day. Those accessing the service between midnight and 7 a.m. are likely to be those working unusual shift patterns. The most popular times of day also coincide with the standard lunch break of a 9 a.m. to 5 p.m. working pattern. Patterns linked with periods of work are also evident when observing the day of week that users access their data, with a clear drop at weekends compared with the standard Monday to Friday “working week”. This may indicate that some users find it more convenient to access their records or only have access to computers during their working time.

The strength of this analysis is that it describes usage of system available to all individuals with diabetes across a complete national population. It also provides a clear comparison of data from the MDMW cohort against national figures to demonstrate the how representative this is when compared with the Scottish diabetes population. As a largely self-selecting cohort, this shows that the MDMW cohort is not fully representative of the background population at this time.

Another limitation is that it is not immediately obvious how the MDMW service could be replicated in environments with more fragmented systems landscapes and complicated data access. The system has been designed in such a way that it can be adapted to link with any other appropriate clinical record via its standardised generic interface. For example, if a suitable data source was available, any organisation would be able to integrate with MDMW, giving their patients the ability to make use of the functionality developed. To demonstrate this, the authors are working with groups in NHS England, and beyond, to implement the service outside Scotland. Making the system more widely available means working closely with a large variety of third-party vendors of systems used to manage diabetes in these environments. This means that new interfacing is required to collect data in a suitably timely manner to make the service up-to-date and accurate for the users. Further publications will follow to describe progress. Finally, this analysis does not describe how patients transfer the information available on MDMW to their daily life and whether frequent access means that the information is fully understood.

To answer these and other questions, a follow-up survey questionnaire was sent to active users in early-2015 to determine their experiences with the service to date [18, 19]. While these results are currently being analysed and will be reported in a separate manuscript, the majority were positive, with some examples of anecdotal feedback received to date are as follows:

*“newly diagnosed and find mdmw very handy as it is near impossible to get through to the doctors these days to get results”*

*“This is superb … I’m so impressed by the information and how it is presented”*

*“What a fab resource, wish we had this in @NHSEngland”*

## Conclusion

My Diabetes My Way is a useful aid to diabetes self-management, providing access to people with diabetes in Scotland. It is unique in offering access to a complete geographical population, providing information from many diabetes-related sources. MDMW supports the diabetes improvement, self-management, healthcare quality and eHealth strategies of the Scottish Government by allowing patients to become more active participants in their care.

Further evaluation will be conducted over the coming months. Analysis will continue to assess impact on clinical outcomes, with preliminary analysis showing improvements amongst those who access the system. Work will continue to expand on these data for peer-reviewed publication.

My Diabetes My Way has potential to be adapted to work with other clinical systems and conditions. We are actively exploring opportunities to roll out the service in regions within NHS England and within Europe and the Middle-East.

## Abbreviations

ePHR: electronic personal health record
MDMW: My Diabetes My Way
NHS: National Health Service
SCI-Diabetes: NHS Scotland’s flagship diabetes electronic medical record
